# Diagnosis of COVID-19 Using Chest X-ray Images and Disease Symptoms Based on Stacking Ensemble Deep Learning

**DOI:** 10.3390/diagnostics13111968

**Published:** 2023-06-05

**Authors:** Abdulaziz AlMohimeed, Hager Saleh, Nora El-Rashidy, Redhwan M. A. Saad, Shaker El-Sappagh, Sherif Mostafa

**Affiliations:** 1 College of Computer and Information Sciences, Imam Mohammad Ibn Saud Islamic University (IMSIU), Riyadh 13318, Saudi Arabia; aialmohimeed@imamu.edu.sa (A.A.); sherif.mostafa@fcih.svu.edu.eg (S.M.); 2 Faculty of Computers and Artificial Intelligence, South Valley University, Hurghada 84511, Egypt; 3 Machine Learning and Information Retrieval Department, Faculty of Artificial Intelligence, Kafrelsheiksh University, Kafrelsheiksh 13518, Egypt; noura.alrashidy@ai.kfs.edu.eg; 4 College of Informatics, Midocean University, Moroni 8722, Comoros; redhwan@midocean.edu.km; 5 Faculty of Computer Science and Engineering, Galala University, Suez 435611, Egypt; 6 Information Systems Department, Faculty of Computers and Artificial Intelligence, Benha University, Banha 13518, Egypt

**Keywords:** machine learning, deep learning, ensemble learning, COVID-19, diagnosis, symptoms, stacking

## Abstract

The COVID-19 virus is one of the most devastating illnesses humanity has ever faced. COVID-19 is an infection that is hard to diagnose until it has caused lung damage or blood clots. As a result, it is one of the most insidious diseases due to the lack of knowledge of its symptoms. Artificial intelligence (AI) technologies are being investigated for the early detection of COVID-19 using symptoms and chest X-ray images. Therefore, this work proposes stacking ensemble models using two types of COVID-19 datasets, symptoms and chest X-ray scans, to identify COVID-19. The first proposed model is a stacking ensemble model that is merged from the outputs of pre-trained models in the stacking: multi-layer perceptron (MLP), recurrent neural network (RNN), long short-term memory (LSTM), and gated recurrent unit (GRU). Stacking trains and evaluates the meta-learner as a support vector machine (SVM) to predict the final decision. Two datasets of COVID-19 symptoms are used to compare the first proposed model with MLP, RNN, LSTM, and GRU models. The second proposed model is a stacking ensemble model that is merged from the outputs of pre-trained DL models in the stacking: VGG16, InceptionV3, Resnet50, and DenseNet121; it uses stacking to train and evaluate the meta-learner (SVM) to identify the final prediction. Two datasets of COVID-19 chest X-ray images are used to compare the second proposed model with other DL models. The result has shown that the proposed models achieve the highest performance compared to other models for each dataset.

## 1. Introduction

The leading cause of the global COVID-19 pandemic is the SARS-CoV-2 virus. Therefore, it has become necessary to find means that would effectively achieve early detection of people with COVID-19 and provide them with the care needed on time. In addition, all medical measures and precautions must be taken to separate patients infected with COVID-19 from other patients to reduce the spread of the disease or its fatal symptoms. The number of deaths due to coronavirus reached 6,517,058 based on global measures [1]. Furthermore, COVID-19 poses a severe challenge due to its ease of transmission and global lack of definitively viable therapies [2]. Many vaccines have been proven to expose users to many complications, including blood clots.

COVID-19 infection goes through three stages: the incubation period, acute COVID-19, and finally, COVID-19 recovery. The incubation period is the period between the actual infection with the disease and the onset of symptoms in the patient. Acute COVID-19 is the time when the symptoms appear, such as fever, cough, fatigue, headache, congestion, or runny nose, among other COVID-19 symptoms. In addition, an essential step in the fight against this fatal illness would be a successful screening and diagnosis procedure to treat affected patients. In addition, an effective strategy in the fight against COVID-19 may be early detection utilizing chest X-ray pictures [3].

Therefore, effective and early COVID-19 diagnosis based on the symptoms and chest X-ray images will help to mitigate the coronavirus outbreak. Moreover, it will assist healthcare systems, including doctors, nurses, and medical staff, in protecting vulnerable patients. Artificial intelligence (AI) has been instrumental in the steady transition from laboratory to clinical and public health applications.

AI provides a wide range of approaches for analyzing complex data to advance understanding of the subject of COVID-19 [4,5,6,7]. AI employs machine learning (ML) and deep learning (DL) to produce algorithms that can be used in the clinical and biomedical fields for patient classification and stratification based on the pairing and processing of a wide range of available data sources, such as heart disease detection [8], polycystic ovary syndrome detection [9], and chronic kidney disease detection [10]. The most significant contribution is using AI to detect patients at higher risk early to treat those patients and control disease transmission. Furthermore, AI can help governments to manage the pandemic by early notification of COVID-19 outbreaks [11]. An ensemble classifier combines the results of several classifiers in a way that enables component models to balance out the deficiencies of each other. Ensemble learning has three types, stacking [12,13], bagging [14], and boosting, which use a generic meta-approach in predictive performance by integrating predictions from different models, improving the general prediction of DL. Stacking involves combining weak algorithms into a meta-model that can make better predictions [12,13].

There are two types of stacking ensembles: heterogeneous and homogeneous. The heterogeneous ensemble uses a variety of classifiers, while the homogeneous ensemble uses the same base model repeatedly. An ensemble of heterogeneous agents may perform better than an ensemble of homogeneous agents because of the combination of their biased decisions. In our work, we develop homogeneous stacking ensemble models to detect COVID-19.

**Figure 1:**
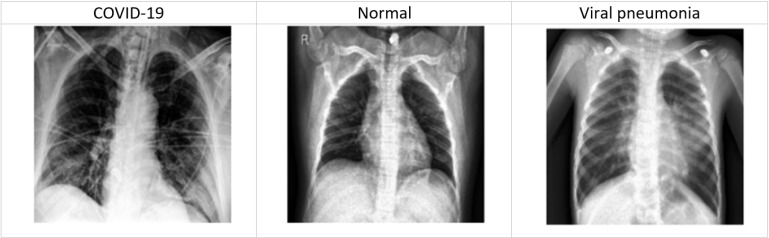
Example of chest X-ray images.

Therefore, rapid diagnosis based on symptoms with accurate prediction is the essential AI-based solution to control the spread of the pandemic. Some related research work has been done on COVID-19 diagnosis. However, experimental research still needs to be done using ensemble learning for COVID-19 diagnosis. On the other hand, COVID-19 can induce pneumonia, which is caused by lung inflammation triggered by bacterial or viral infection. Consequently, researchers, specialists, and companies have used medical images (i.e., chest X-ray and computed tomography (CT)) for early diagnosis of COVID-19 patients. Hundreds of chest X-ray have been used to investigate the nature of pneumonia due to COVID-19 infection (see Figure 1). According to the context of this paper, DL models have been proposed to study chest X-ray images to benefit the detection of COVID-19. Researchers have used DL classifiers, in particular, to classify COVID-19 using chest X-rays images. For example, CNN models have been used to learn the pattern of COVID-19 infection from radiological X-ray images [15,16]. In particular, CNN models help to draw a clear distinction between non-tangible elements in the X-ray that can expose COVID-19 infection.

In our work, we develop homogeneous stacking ensemble models to detect COVID-19 based on symptoms and chest X-ray images. Our contributions can be summarized as follows:

- We propose two stacking ensemble DL models to detect COVID-19 using symptoms and chest X-ray images.
- The first proposed model is merged from the outputs of pre-trained DL models in the stacking: MLP, RNN, LSTM, and GRU; it uses stacking to train and evaluate the meta-learner (SVM) to identify the final prediction based on symptoms.
- The second proposed model is merged from the outputs of pre-trained models in the stacking: ResNet152V2, DenseNet201, VGG16, MobileNetV2, and inception_v3i; it uses stacking to train and evaluate the meta-learner (SVM) to identify the final prediction based on chest X-ray images.
- The first proposed model is evaluated against MLP, RNN, LSTM, and GRU using two COVID-19 symptom datasets and different assessment techniques: accuracy (A), recall (R), precision (P), and f1-score (F1).
- The second presented model is compared to ResNet152V2, DenseNet201, VGG16, MobileNetV2, and inceptionv3i utilizing COVID-19 chest X-ray images and different assessment techniques.
- A comparison of the proposed models with other models shows that the proposed models have the highest performance.

The study’s structure is as follows: Section 2 discusses COVID-19 detection based on symptoms and chest X-ray images. The methodology and proposed models are discussed in Section 3. In Section 4, the experimental results are depicted. Conclusions are presented in Section 6.

## 2. Related Work

This section presents recent studies on the subject of detecting COVID-19 using symptoms and chest X-ray images.

### 2.1. Detecting COVID-19 Using Symptoms

The authors used ML and DL algorithms to detect COVID-19. For example, in [6], the authors used the gradient-boosting (GBoost) model for COVID-19 patient detection. They evaluated the model using AUC. In [17], the authors proposed a DL model technique called gray level co-occurrence matrix (GLCM) based on CNN. The authors in [18] contrasted widely employed feature extraction techniques for COVID-19 automatic categorization based on DL. The authors applied a group of deep CNNs, including InceptionV3, InceptionResNetV2, MobileNet, DenseNet, Xception, ResNet, VGGNet, and NASNet. In [19], the authors developed a predictive algorithm based on a trained DL model using 8427 COVID-19 patient records. In [20], the authors used the ML models: XBoost, AdaBoost, RF, and ExtraTrees with 337 COVID-19 patients. Jamshidi et al. [21] summarized different models, including hybrid DL approaches and ML approaches, for calculating and forecasting complicated occurrences focused on the spread of COVID-19. In [22], the authors used ML techniques to detect mortality risks in COVID-19 using a dataset collected from the UK Biobank. The authors of [23] used KNN, SVM, LR, multilayer perceptual neural networks (MLP), LSTM, and GRU for COVID-19 diagnosis. They used the COVID-19 dataset from Kaggle [24] that includes some features and symptoms for their experiment. In [25], the authors used LR, NB, RF, DT, and gradient boosters for COVID-19 diagnosis based on some symptoms. They used the COVID-19 dataset from Kaggle [24] that includes some features and symptoms. The results showed that KNN achieved the highest accuracy. In [26], the authors Used RF, SVM, MLP and XGB, and LR to predict COVID-19 for children based on collected data that include some of the symptoms.

Previous studies used regular ML and DL models. However, they did not use ensemble stacking based on LSTM and GRU. In our study, we proposed stacking ensemble DL models for detecting COVID-19. The proposed model combined LSTM and GRU with SVM as a meta-learner for detecting COVID-19.

### 2.2. The Detection of COVID-19 Using Chest X-rays

Several studies have used transfer learning on chest X-ray images to identify COVID-19 patients. Here, we focus only on issues directly relevant to our suggestion.

In [27], X-ray images of the chest were analyzed using three pre-trained models for extracting features and detecting COVID-19. A variety of data augmentation techniques, such as random rotation and noise, were employed. VGG16 achieved the best results.

In [28], A total of 100 chest X-ray images was analyzed by the author to detect COVID-19 using three pre-trained CNNs, Inception-ResNetV2, InceptionV3, and ResNetV2. ResNet50 registered the highest result. In [29], the authors proposed CNN models (COVID-Net) and proposed a new design pattern called residual projection extension-projection extension (PEPX).

The authors of [30] proposed a concatenation-based CNN (Concat_CNN) model to detect COVID-19 from chest X-rays images. A comparison was made between Concat_CNN and the following transfer models: VGG16, InceptionV3, Resnet50, and DenseNet121. Concat_CNN registered the best results.

In [31], the authors suggested a CNN employing Softmax classifier and ML (SVM and RF).

In [32], the authors presented a hybrid CNN model using Xception and ResNet101 to extract COVID-19 characteristics from chest X-rays.

In [33], the authors proposed new ML models to detect COVID-19 from chest X-ray images. They used fractional multichannel exponent moments to extract features from images. In [34], the authors presented a DL model and employed SqueezNet with a modified output layer to categorize X-ray pictures into COVID-19, normal, and pneumonia. In [35], the authors developed deep CNN (DCNN) to detect COVID-19 with five classes and compared it with eight pre-trained models. Based on the results, DCNN had the highest accuracy.

In [36], the authors used VGG16, VGG19, DenseNet201, Inception_ResNet_V2, Inception_V3, Resnet50, and MobileNet_V2 with five classes.

Table 1 provides an overview of previous studies on COVID-19 detection.

## 3. Materials and Methods

Our work aims to develop the proposed stacking ensemble models for detecting COVID-19 using two data types: chest X-ray images and some of the symptoms. This section describes the methodology and the framework of stacking ensemble models based on two data types.

### 3.1. Detecting COVID-19 Based on Symptoms

**Figure 2:**
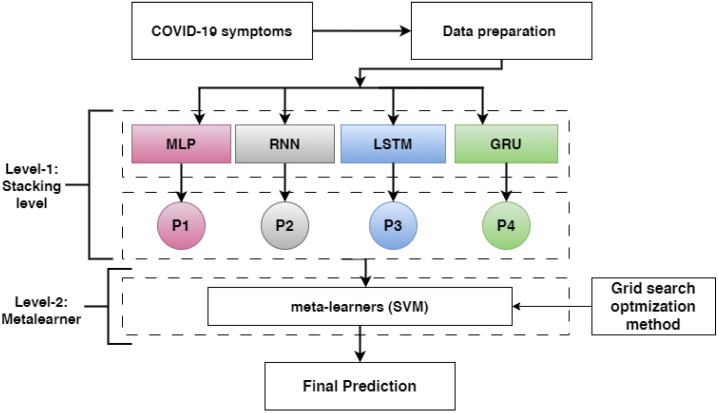
The proposed method for detecting COVID-19 based on symptoms (P1, P2, P3, and P4 refer to the probability outputs of each model).

This subsection describes the proposed methods to detect COVID-19 based on symptoms, as shown in Figure 2. First, two symptoms included in the COVID-19 datasets are described. Second, the data splitting is presented. Third, the DL model architecture, including MLP, RNN, LSTM, and GRU, and optimization methods for DL models are presented. Finally, we discuss how the DL pre-trained models were combined using stacking ensemble learning techniques.

#### 3.1.1. COVID-19 Symptoms Dataset Description

Two datasets of COVID-19 symptoms are used to conduct our experiment.

- The first dataset of symptoms of COVID-19 (COVID-19-Symptoms-1) is downloaded from GitHub [37], and it includes 13 features and one class label. The class label has 755 recovered (0) and 108 deaths (1); 250 rows for class 0 are selected. Based on some pre-defined standard symptoms, the data will help determine whether a person will recover from COVID-19 symptoms. WHO guidelines are used to determine these symptoms. An explanation of the features of COVID-19-Symptoms-1 is described in Table 2.

**Table 2:** Description of the features of COVID-19-Symptoms-1.

Features	Descriptions
location	What region of the country
country	The place where the person lives
gender	Male or female
age	Age of patient
vis_wuhan	Indication of whether the person has visited Wuhan
from_wuhan	Whether the person is from Wuhan, China, or not
symptom1, symptom2, symptom3, symptom4, symptom5 and symptom6	Six features of symptoms
diff_sym_hos	Time before symptoms appear
result	Recovered or death

**Figure 3:**
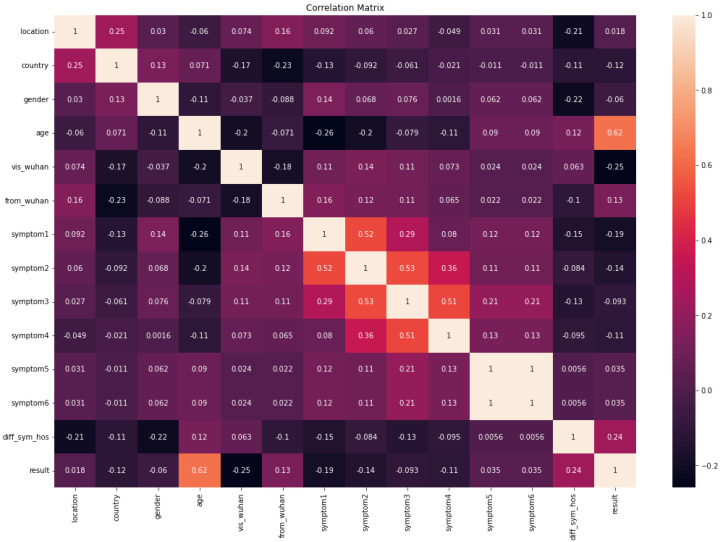
Correlation matrix of COVID-19-Symptoms-1.

Figure 3 shows the correlation matrix of the COVID-19-Symptoms dataset. We can see that the symptoms are highly correlated with each other. Age and diff_sym have the highest correlation with the results.

- The second dataset of COVID-19 symptoms (COVID-19-Symptoms-2) [24] covers the presence of several features (mask use, trip overseas, and interaction with a COVID patient), as well as multiple symptoms (fever, dry cough, and breathing issues); in addition, the class label refers to whether the person has COVID or not. There are 4347 rows for the training set, and 1087 for the testing set. A description of the features of COVID-19-Symptoms-2 is shown in Table 3.

**Table 3:** Description of the features of COVID-19-Symptoms-2.

Features	Description
BP	Difficulty in breathing due to breathing problems Ranges from mild, to moderate, to severe
F	Increase in patient temperature (commonly over 38)
DC	Type of cough that usually does not produce any phlegm
ST	A common symptom that is mainly characterized by pain or itchiness in the throat
RN	Discharge of fluid due to viral or bacterial causes
AS	Chronic respiratory diseases usually narrow the airway path and cause breathing problems
CLD	A medical condition that causes long-term problems with breathing
HD	Felling pain or discomfort in the face region, ranges from mild, to moderate, to severe
Heart	A medical condition that affects blood vessels and heart status
DI	Chronic diseases in which the patient becomes unable to produce sugar at a regular level due to pancreas problems
HT	Chronic diseases in which the force of the blood against the direction of the walls is higher than normal
FA	The feeling of pain or illness due to extreme effort or tiredness
GA	This refers to the digestive system where all processing and absorbing of food occurs
AL	Check if the patient traveled in the last 14 days
CW	Direct connection with positive cases of COVID-19
AL	Check if the patient has attended recent gatherings (i.e., festival, party)
VP	Check if the patient has visited an exposed place
FW	If any one of the patient’s family works in one of the exposed places
WM	Check if the patient’s mask wearing continues when outside
SF	Check if the patient visits a place for sanitization
COVID-19	The final decision (yes for positive, no for negative)

**Figure 4:**
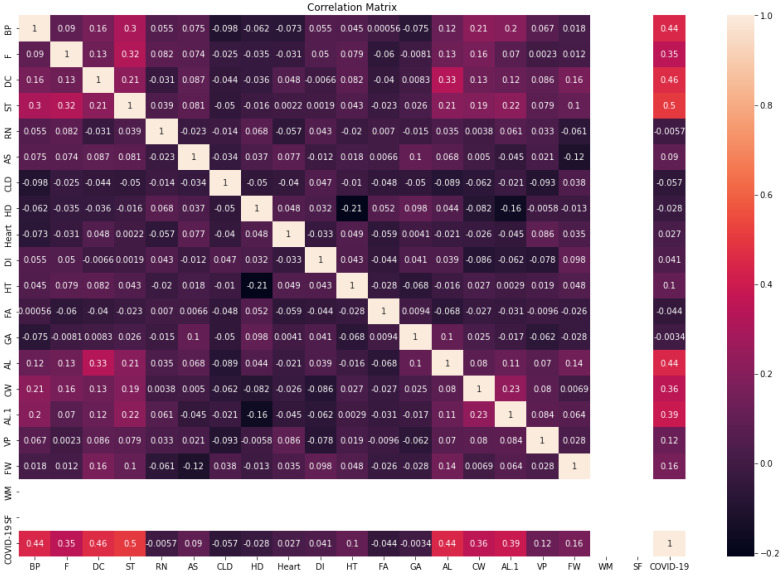
Correlation matrix of COVID-19-Symptoms-2.

Figure 4 shows the correlation matrix of the COVID-19-Symptoms dataset2. We can see that the WM and SF features have one value; therefore, they are removed from the dataset. In addition, we transformed categorical features into numerical data using LabelEncoder in Python.

#### 3.1.2. Data Splitting

Datasets are divided 80/20 into training and testing sets. A training set was used for training and optimizing models, and a testing set was used for evaluating models. Additionally, 10% of training sets are used as validation sets.

Table 4 presents the number of rows for each class in COVID-19-Symptoms-1 and COVID-19-Symptoms-2, respectively.

#### 3.1.3. DL Models

The MLP, RNN, LSTM, and GRU are trained and evaluated in accordance with our objective datasets. The final layer of each model includes three neurons and a softmax function; the loss function is categorical cross-entropy, and the optimizer is Adam [38].

- A multilayer perceptron (MLP) is a neural network that complements forward neural networks. It has three layers: input, output, and hidden. The input layer receives input signals [39].
- Recurrent neural networks (RNN) keep a state vector in their hidden units that indirectly provides information about the history of all previous items in an input sequence [40]. A basic RNN contains three layers: input, recurrent hidden, and output. N input units are present in the input layer. This layer’s inputs are a series of vectors traversing time t [41]. The input units in the hidden layer are fully linked to the hidden units in the hidden layer, with the connections determined by a weight matrix. The hidden layer includes M hidden units, which are linked together in time via recurrent connections [42].
- Long short-term memory (LSTM) architecture is applied to DL algorithms as an attention-based RNN. LSTMs have feedback connections. A complete data sequence can be analyzed, as well as single data points. In LSTM mode, one of the most crucial components is the “cell state” of the memory cell, which maintains its state over time [43].
- Gated recurrent units (GRUs), a type of RNN, use gate units to control information flow rather than separate memory cells. GRUs contain two gate operating mechanisms to solve the challenge posed by standard RNNs: an update gate and a reset gate [44,45]. The update gate ensures that the necessary memory is retained in order to go to the next stage. In order to advance to the next stage, the update gate ensures that enough memory is retained. The reset gate controls how previously stored data are updated with a new input. After the reset gate engages, a newer memory content is created for the details of the preceding time step [46].

#### 3.1.4. Optimization Methods

Hyperparameter tuning is the process of adapting hyperparameters to obtain the right set of values that optimizes the performance of a DL. A hyperparameter is a variable that determines the training process and model topology for DL models. These variables directly impact DL performance throughout the training process. KerasTuner [47] is a Python library explicitly developed for tuning DL hyperparameters. KerasTuner supports different types of algorithms, namely Bayesian optimization, hyperband, Sklearn, and random search [47]. Some hyperparameters are adapted, such as the number of units (ranging between 20 and 800) and the width of hidden layers.

#### 3.1.5. The Proposed Model

Stacking is combining the different models’ output with training other models to produce the best result. Heterogeneous stacked ensemble is a strategy for blending many heterogeneous models by learning by meta-learner to predict the final results [48]. The idea behind stacking is that some models will fit the categories of a test observation properly while others will not [49]. The algorithm learns from the variety of predictions and seeks to integrate the models to improve the performance of the basic models [50].

Two levels are proposed in our model: level-1 and level-2.

- In level-1, each base-learner (MLP, RNN, LSTM, and GRU) is trained separately and saved. Then, the pre-trained models (RNN, LSTM, and GRU) are loaded, and all layers are frozen without the output layer. Each model takes a training set and predicts the training output of a probability (p1, p2, and p3). Then, the training outputs are combined in stacking, which is called training stacking.
- In level-2, the meta-learner (SVM) is trained and optimized using training stacking. The meta-learner (SVM) is evaluated and tested using testing stacking to predict the final results. The meta-learner is optimized using a grid search with different parameter values.

### 3.2. Detecting COVID-19 Based on the Dataset of Chest X-ray Images

**Figure 5:**
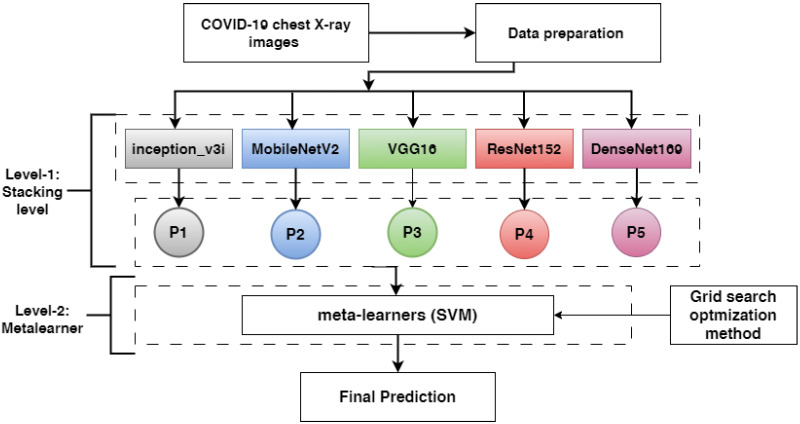
The proposed method for detecting COVID-19 based on chest X-ray images (P1, P2, P3, P4, and P5 refer to the probability outputs of each model).

This subsection describes the proposed methods to detect COVID-19 based on symptoms. First, chest X-ray images are described. Second, the data preparation procedure involving data augmentation and image resizing is presented. Third, the pre-trained models ResNet152V2, DenseNet201, VGG16, MobileNetV2, and inception_v3i are presented. Finally, we discuss how the pre-trained models were combined using stacking ensemble learning techniques. Figure 5 shows the proposed methodology’s overall workflow in detail.

#### 3.2.1. COVID-19 Chest X-ray Images Description

- COVID-19 -chest-X-ray-1

Kaggle provided 317 chest X-ray images [51] in three classes: 137 images with COVID-19, 90 images with normal imaging, and 90 images with viral pneumonia. A total of 251 images are available for training and 66 images are available for testing.

- COVID-19-chest-X-ray-2

A total of 2060 CHX-Ray images were downloaded from Kaggle [52]. Of these, 696 images were selected for testing and 2060 for training.

#### 3.2.2. Data Augmentation

Preprocessing the first X-ray chest images is required to enhance image features and improve image data quality. First, RGB is modified for the image channel sequence. Second, these images are resized to 224 × 224 × 3. Third, image augmentation is performed, which is a method of producing additional dataset points from existing data by developing changed copies of a dataset [53,54,55]. A variety of augmentation strategies are applied: rescale:1./255, zoom_range:0.1, rotation_range:20, width_shift_range:0.1, height_shift_range:0.1, and horizontal_flip:True.

#### 3.2.3. Fine-Tuning the Pre-Trained

The pre-trained ResNet152V2, DenseNet201, VGG16, MobileNetV2, and inception_v3i are picked and fine-tuned in accordance with our objective datasets. The final layer of each model includes three neurons and a softmax function; the loss function is categorical cross-entropy, and the optimizer is Adam.

- Visual geometry group (VGG): In a convolutional neural network architecture, Zisserman and Simonyan proposed VGG in 2014 [56]. The essential part of this architecture is that rather than depending on a huge number of hyperparameters, it concentrates on fundamental size kernels in the convolutional layers and kernels in the max-pooling layers. In the end, there are two fully connected layers, followed by a softmax for output [57,58]. VGG19 differs from VGG16 in that it contains an extra layer in the three convolutional blocks [59].
- Densely connected convolutional networks (DenseNet): The dense convolutional network recognizes the input image size, which uses dense connections across layers with dense blocks. The network spans 201 layers of depth while connecting all layers directly with each other with feed-forward using matching feature-map sizes [60]. Each layer receives extra inputs from all previous levels and relays its feature maps to all previous layers to maintain the system’s feed-forward structure. Compared to conventional networks, DenseNet can outperform ordinary networks by increasing processing needs, reducing parameter count, increasing feature reuse, and maintaining feature propagation [61].
- Deep residual networks (ResNet) employ residual blocks to increase model accuracy for image classification. The skip connections are crucial to the residual blocks and the strength of this form of neural network [62]. One residual block consists of a convolution layer preceded by a batch normalization layer that adjusts to retain a mean outcome closer to 0. The output standard deviation is near one, and a ReLU activation function is used. This is followed by a convolution layer and a batch normalizing layer [63]. The skip connection bypasses both levels and is added immediately before the ReLU activation function. Such residual blocks are repeated to construct a residual network. ResNet comes in a variety of forms that all follow the same basic idea but employ different numbers of layers [64]. It has five stages, each with a convolution and identity block, and each convolution and identity block has three convolution layers [65].
- The inception network, a significant landmark in the creation of CNN classifiers, incorporates a block of parallel convolutional layers with three distinct filter sizes [66]. In addition, max pooling is conducted. Because of the varying filter sizes, the network has the ability to learn multiple variabilities at different scales using convolutions [67]. Concatenated results are forwarded to the following conception module [68]. The max-pooling layer in an inception module may benefit from padding to keep its height and breadth consistent with the other outputs (feature maps) of the convolutional layers in the same inception module [69].
- Xception is a 71-layer deep convolutional neural network that has an input image size of 299 upon swapping the normal inception modules with depthwise separable convolutions [66,70]. Depthwise separable convolution layers are based on the principle that convolutional neural network feature maps resulting from such cross-channel and spatial correlation translation could be entirely independent [71].
- MobileNet is a simplified design that employs depthwise separable convolutions created by mixing two 1D convolutions with two kernels to generate lightweight deep convolutional neural networks [72]. This means that less memory and fewer parameters are required for training, resulting in a more efficient model for mobile and embedded vision applications [73].

#### 3.2.4. The Proposed Model

Two levels are proposed in our model: level-1 and level-2.

- In level-1, each base-learner (ResNet152V2, DenseNet201, VGG16, MobileNetV2, and inception_v3i) is trained separately and saved. Then, the pre-trained models (ResNet152V2, DenseNet201, VGG16, MobileNetV2, and inception_v3i) are loaded, and all layers are frozen without the output layer. Each model takes a training set and predicts a training probability output (p1, p2, p3,p4, and p5). Then, the training outputs are combined in stacking, which is called training stacking.
- In level-2, the meta-learner (SVM) is trained and optimized using training stacking. The meta-learner (SVM) is evaluated and tested using testing stacking to predict the final results. The meta-learner is optimized using a grid search with different parameter values.

## 4. Experiments Results

This section describes the results of testing DL models and the proposed models using two COVID-19 symptom datasets and two chest X-ray image datasets to detect COVID-19.

### 4.1. Experiment Setup

The experiments were conducted with Python using Google Colab. The Scikit-learn package was used for ML, while the Keras library was used for DL.

### 4.2. Evaluation

The evaluation metrics were applied to assess the learning algorithms. The following four metrics were used to assess classification performance: accuracy (A), precision (P), recall (R), and F1-score (F1).

- Accuracy is a popular evaluation parameter for classification problems. It is the proportion of correct forecasts relative to total predictions [74].
  (1) $$Accuracy=\frac{TP+TN}{TP+FP+TN+FN}.$$
- Precision is a measure for determining categorization accuracy. The equation represents the proportion of correct positive classifications relative to total anticipated positive classifications [74].
  (2) $$Precision=\frac{\mathrm{TP}}{\mathrm{TP}\;+\;\mathrm{FP}}$$
- Recall is the number of accurately detected positive cases out of the total number of positive cases. Returning to the fraud issue, the recall value will be quite valuable in fraud scenarios. A high recall value indicates that a significant number of fraud cases are recognized in comparison to the total number of frauds [74].
  (3) $$Recall=\frac{TP}{TP+FN}$$
- The F1-score measures the mean of the model’s precision and recall [74].
  (4) $$F1\text{-}score=\frac{2\cdot precision\cdot recall}{precision+recall}$$

True positive (TP), true negative (TN), false positive (FP), and false negative (FN) values were recorded. A TP indicates the set of correctly formed positive values, a FP indicates the number of negative values generated incorrectly, a TN indicates the number of negative values generated correctly, and a FN indicates the number of positively predicted values that were correctly created.

### 4.3. Results COVID-19 Symptoms Datasets

This section explores the ability of our proposed model to detect COVID-19 based on symptom datasets.

#### 4.3.1. Parameters Configuration

A training set is used to optimize and train DL models. Some parameters were adopted in RNN, LSTM, and GRU to conduct experiments, such as batch_size = 200 and epoch = 50 with a learning rate of 0.0001, and Adam optimizer. In addition, we used KerasTuner to optimize some parameters in RNN, LSTM, and GRU. The final values of parameters for each model are shown in Table 5.

#### 4.3.2. COVID-19-Symptoms-1

Table 6 shows the results of DL models, as well as the proposed model, using COVID-19-Symptoms-1. We can observe that Proposed-Layer2 achieved the highest performance compared to other models.

Regarding DL models with one layer, Among the four metrics, RNN-Layer1 scored the lowest: 84.72, 84.31, 84.72, and 83.98, respectively. According to the evaluation metrics, the second best results were obtained by MPL-Layer1: 93.06, 93.66, 93.06, and 92.72.

Regarding DL models with two layers, RNN-Layer2 recorded the lowest results in several metrics: 87.5, 87.43, 87.5, and 86.89 in terms of A, P, R, and f1, respectively. The second-best results were obtained from MLP-Layer2 according to different evaluation metrics: 94.44, 94.77, 94.44, and 94.52 in terms of A, P, R, and f1, respectively. Proposed-Layer2 improved performance in different metrics: A by 2.45, P by 2.38, R by 2.45, and f1 by 2.54 compared to MLP-Layer2.

#### 4.3.3. COVID-19-Symptoms-2

Table 7 shows the results of DL models, as well as the proposed model, using COVID-19-Symptoms-2. We can observe that Proposed-Layer2 achieves the highest performance compared to other models.

Regarding DL models with one layer, LSTM-Layer1 recorded the lowest results in several metrics: 94.44, 94.77, 94.44, and 94.52. The second-best results were obtained from MPL-Layer1 according to different evaluation metrics: 97.52, 97.56, 97.52, and 97.53. Proposed-Layer1 improved performance by several metrics: A by 0.78, P by 0.76, R by 0.78, and f1 by 0.77 compared to MPL-Layer1.

Regarding DL models with two layers, LSTM-Layer2 records the lowest results in the different metrics: 96.87, 96.84, 96.87, and 96.85. The second-best results were obtained from MPL-Layer2 according to different evaluation metrics: 98.10, 98.10, 98.10, and 98.10. Proposed-Layer2 improved performance in several metrics: A by 1.20, P by 1.20, R by 1.20, and f1 by 1.21 compared to MPL-Layer2.

### 4.4. Results of Chest X-ray Images Datasets

This section explores the ability of our proposed model to detect COVID-19 based on chest X-ray datasets.

#### 4.4.1. Parameters Configuration

For training ResNet152V2, DenseNet201, VGG16, MobileNetV2, and inception_v3i, some parameters were adopted to conduct experiments, such as batch_size = 64 and epoch = 100 with a learning rate of 0.001 and Adam optimizer. The activation function is softmax, and the loss function is categorical cross-entropy.

#### 4.4.2. COVID-19-Chest-X-ray-1

Table 8 shows the results of models, including the proposed model, using COVID-19-chest-X-ray-1. Comparing the proposed model to other models, it was the most efficient. The proposed model improved performance in several metrics: A by 1.38, P by 1.4, R by 1.38, and F1 by 1.38 compared to ResNet152V2 and VGG16.

ResNet152V2 and VGG16 recorded similar performance in terms of different metrics: 98.24, 98.24, 98.26, and 98.24. The third-highest results were obtained from MobileNetV2 according to evaluation metrics: 96.97, 96.97, 96.97, and 96.97. Finally, inception_v3i recorded the lowest scores: 93.94, 94.08, 93.94, and 93.98.

#### 4.4.3. COVID-19-Chest-X-ray-2

Table 9 shows the results of models, including the proposed model, using COVID-19-chest-X-ray-2. Comparing the proposed model to other models, it was the most efficient. The proposed model improves performance in different metrics: A by 2.22, P by 2.29, R by 2.22, and F1 by 2.21 compared to MobileNetV2.

MobileNetV2 recorded the second-highest performance in different metrics: 96.26, 96.27, 96.26, and 96.27 in terms of A, P, R, and F1, respectively. The third-highest model was obtained from VGG16 according to the evaluation metrics: 95.55, 95.56, 95.55, and 95.55. Finally, inception_v3i recorded the lowest scores: 93.25, 93.25, 93.25, and 93.24.

## 5. Discussion

This section presents the best models of the COVID-19 symptoms dataset and COVID-19 chest X-ray images. It also shows a comparison between the proposed model and recent studies.

### 5.1. COVID-19 Symptoms Datasets

#### 5.1.1. The Best Models for COVID-19 Symptoms Datasets

A stacking ensemble model was proposed to detect COVID-19 sickness by combining the pre-trained models MLP, RNN, LSTM and GRU with the meta-learner model SVM. The proposed model achieved the highest performance in the two datasets compared to other models.

**Figure 6:**
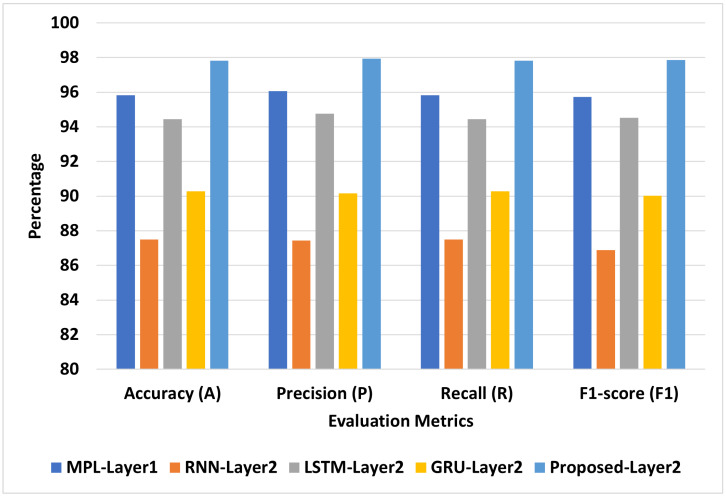
The best models for COVID-19-Symptoms-1.

Figure 6 presents the best models for COVID-19-Symptoms-1. We can see that Proposed-Layer2 recorded the highest scores using different matrices: A = 98.28, P = 98.44, R = 98.28, and F1 = 98.26. MLP-Layer2 recorded the second-highest scores using different matrices: A = 95.83, P = 96.06, R = 95.83, and F1 = 95.72. RNN-Layer2 recorded the lowest A, P, R, and F1 at 87.5, 87.43, 87.5, and 86.89, respectively.

**Figure 7:**
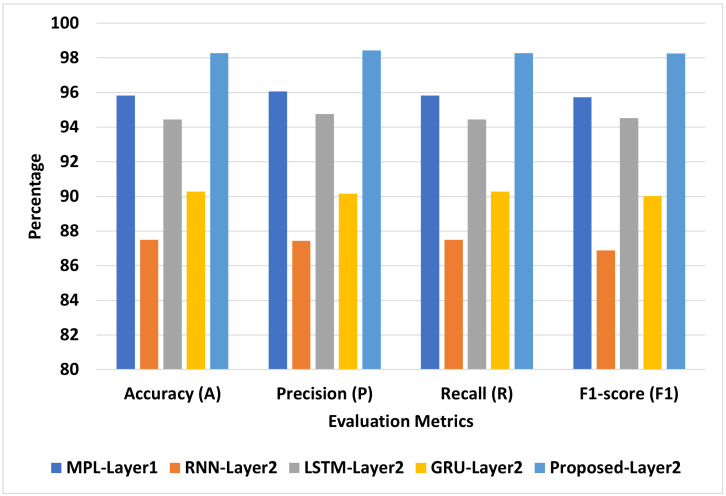
The best models for COVID-19-Symptoms-2.

Figure 7 presents the best models for COVID-19-Symptoms-2. We can see that Proposed-Layer2 recorded the highest scores using different matrices: A = 99.30, P = 99.30, R = 99.30, and F1 = 99.31. MLP-Layer2 recorded the second-highest scores using different matrices: A = 98.10, P = 98.10, R = 98.10, and F1 = 98.10. LSTM-Layer2 recorded the lowest A, P, R, and F1 at 96.87, 96.84, 96.87, and 96.85, respectively.

#### 5.1.2. Comparison with Literature Studies for COVID-19 Symptoms Dataset

Table 10 shows a comparison of previous studies that used COVID-19- Symptoms-2 [24] with the proposed models. In [23], the authors used GRU, which recorded A = 98.65, R = 98.6, P = 99.2, and F1 = 99.2. In addition, in [25], the authors used KNN, which registered A = 97.97, R = 97.97, P = 97.97, and F1 = 97.97. The proposed model achieved the highest performance compared to [23,25].

### 5.2. COVID-19 Chest X-ray Images Datasets

A stacking ensemble model was proposed to detect COVID-19 sickness by combining the pre-trained models ResNet152V2, DenseNet201, VGG16, MobileNetV2, and inception_v3i with the meta-learner model SVM. The proposed model achieved the highest performance in the two datasets compared to other models.

#### 5.2.1. The Best Models for Chest X-ray Image Datasets

This section presents the best models used with chest X-ray image datasets.

**Figure 8:**
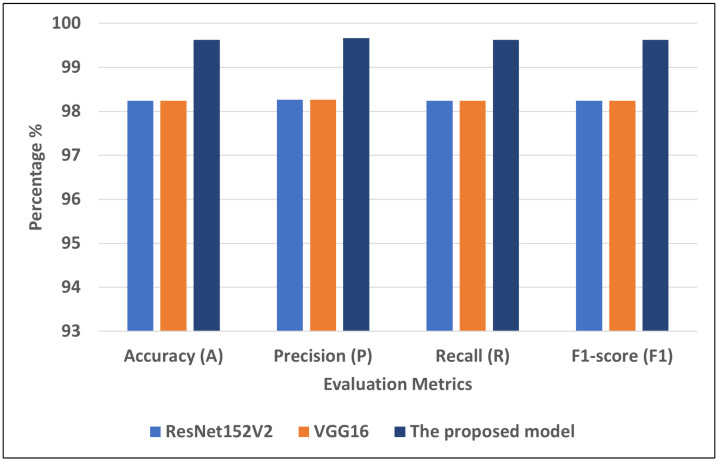
The best models for COVID-19-chest-X-ray-1.

Figure 8 presents the best models for COVID-19-chest-X-ray-1. We can see that the proposed model recorded the highest scores using different matrices: A = 99.62, P = 99.66, R = 99.62, and F1 = 99.62. VGG16 and ResNet152V2 recorded the second-highest scores using different matrices: A = 98.24, P = 98.26, R = 98.24, and F1 = 98.24.

**Figure 9:**
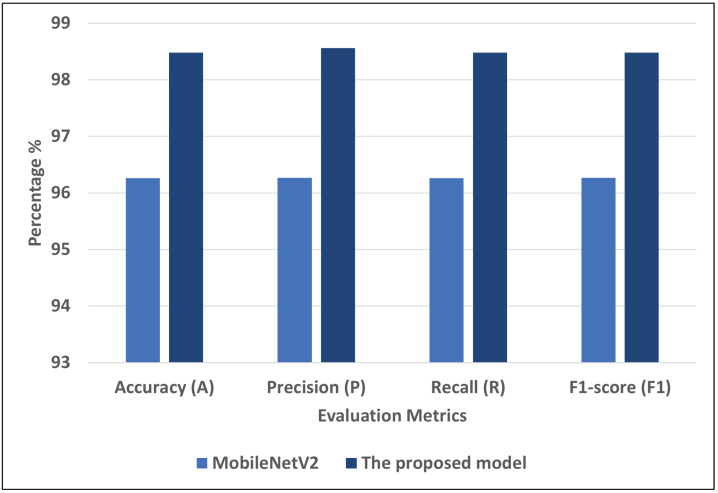
The best models for COVID-19-chest-X-ray-2.

Figure 9 represents the best models for COVID-19-chest-X-ray-2. We can see that the proposed model recorded the highest scores using different matrices: A = 98.48, P = 98.56, R = 98.48, and F1 = 98.48. MobileNetV2 record the second-highest scores using different matrices: A = 96.26, P = 96.27, R = 96.26, and F1 = 96.27.

#### 5.2.2. Comparison with Literature Studies

Table 11 shows a comparison between the proposed model and recent studies using COVID-19-chest-X-ray-2 with two or three classes. We can see that the proposed model achieved the highest performance. The authors detected COVID-19 results in three classes: COVID-19, normal, and pneumonia. In [28], ResNet50 recorded A = 98 and R = 96.46. In [29], the authors indicated that COVID-Net has a recorded accuracy at 92.4 A. In [30], the authors proposed Concat_CNN, which recorded A = 96.31, P = 95.8, and R = 92.99. In [32], a concatenated CNN model was proposed and recorded A = 98.02, F1 = 98.24, P = 97.04, and R = y. The authors of [34] used SqueezNet, with recorded accuracy of A = 95, P = 94.66, R = 94.66, and F1. In [3], XGBoost recorded A = 97.87, P = 97.87, and R.

## 6. Conclusions

This paper proposes a stacking ensemble DL model using COVID-19 symptoms and chest X-ray images to detect the disease. Two models have been proposed for use with the different datasets, including one based on COVID-19 symptoms and one based on chest X-ray images. The first proposed model combines four pre-trained deep learning models, MLP, RNN, LSTM, and GRU, together into a stacking so that a meta-learner is trained and evaluated to identify a final prediction. In comparison to DL models based on two COVID-19 symptom datasets, our proposed model achieved the highest performance (A = 99.30, P = 99.30, R = 99.30, and F1 = 99.31). The second proposed model has merged the outputs of the pre-trained models ResNet152V2, DenseNet201, VGG16, MobileNetV2, and inception_v3i in a stacking and uses stacking to train and evaluate the meta-learner (SVM) to identify the final prediction using chest X-ray datasets. Comparing the proposed model to DL models based on the two COVID-19 chest X-ray datasets, it achieved the best performance (A = 99.62, P = 99.66, R = 99.62, and F1 = 99.62). Our proposed models were applied to two different types of datasets, COVID-19 symptoms and chest X-ray images, and it achieved the highest performance in measuring the generalizability of the proposed model. However, our model needs some enhancements, which will be considered in future work, including (1) testing the model on other datasets and (2) applying explainable AI (XAI).

## Figures and Tables

**Table 1:** Summary of previous studies on the detection of COVID-19.

Papers	Methods	Datasets
[6]	GBoost	51,831 tested individuals from Israeli Ministry of Health public dataset
[17]	GLCM based on CNN	Chest X-rays (273 X-rays, frontal view)
[18]	Different types of pretrained CNN	Kaggle’s chest X-ray images (pneumonia)
[20]	X_GBoost, AdaBoost, RF, and ExtraTrees	337 COVID-19-positive patients at Cheikh Zaid Hospital
[22]	Data-driven RF	11,245 participants in UK from Biobank dataset
[23]	KNN, SVM, LR, MLP, LSTM, and GRU	COVID-19-Symptoms-2
[25]	RF, SVM, MLP, and XGB	COVID-19-Symptoms-2

**Table 4:** The number of rows in COVID-19 datasets.

Datasets	Classes	Training Set	Testing Set
COVID-19-Symptoms-1	Recovered	198	52
	Death	88	20
	Total	286	72
COVID-19-Symptoms-2	Yes	3501	882
	No	846	205
	Total	4347	1087

**Table 5:** In order to detect COVID-19 based on symptoms, the best parameter values were selected for RNN, LSTM, and GRU.

Datasets	Models	Number of Units
COVID-19-Symptoms-1	RNN-Layer1	[490]
	RNN-Layer2	[690,450]
	LSTM-Layer1	650
	LSTM-Layer2	[350,370]
	GRU-Layer1	530
	GRU-Layer1	[530,310]
COVID-19-Symptoms-2	RNN-Layer1	[570]
	RNN-Layer2	[430,330]
	LSTM-Layer1	[230]
	LSTM-Layer2	[570,350]
	GRU-Layer1	[250]
	GRU-Layer1	[610,570]

**Table 6:** The A, P, R, and F1 of applying models to COVID-19-Symptoms-1.

Models	Models	Accuracy	Precision	Recall	F1-Score
DL models	MPL-Layer1	93.06	93.66	93.06	92.72
	MPL-Layer2	95.83	96.06	95.83	95.72
	RNN-Layer1	84.72	84.31	84.72	83.98
	RNN-Layer2	87.5	87.43	87.5	86.89
	LSTM-Layer1	91.67	91.56	91.67	91.53
	LSTM-Layer2	94.44	94.77	94.44	94.52
	GRU-Layer1	90.28	90.55	90.28	89.81
	GRU-Layer2	90.28	90.17	90.28	90.02
The proposed model	Proposed-Layer1	96.83	96.81	96.83	96.80
	Proposed-Layer2	98.28	98.44	98.28	98.26

**Table 7:** The A, P, R, and F1 of applying models to COVID-19-Symptoms-2.

Approaches	Models	A	P	R	F1
DL models	MPL-Layer1	97.52	97.56	97.52	97.53
	MPL-Layer2	98.10	98.10	98.10	98.10
	RNN-Layer1	97.33	97.31	97.33	97.31
	RNN-Layer2	97.61	97.62	97.61	97.61
	LSTM-Layer1	94.44	94.77	94.44	94.52
	LSTM-Layer2	96.87	96.84	96.87	96.85
	GRU-Layer1	96.6	96.55	96.6	96.55
	GRU-Layer2	97.24	97.25	97.24	97.25
The proposed model	Proposed-Layer1	98.30	98.32	98.30	98.30
	Proposed-Layer2	99.30	99.30	99.30	99.31

**Table 8:** The A, P, R, and F1 of applying models to COVID-19-chest-X-ray-1.

Models	A	P	R	F1
ResNet152V2	98.24	98.26	98.24	98.24
DenseNet201	96	96	96	96
VGG16	98.24	98.26	98.24	98.24
MobileNetV2	96.97	96.97	96.97	96.97
inception_v3i	93.94	94.08	93.94	93.98
The proposed model	99.62	99.66	99.62	99.62

**Table 9:** The A, P, R, and F1 of applying models to COVID-19-chest-X-ray-2.

Models	A	P	R	F1
ResNet152V2	95.11	95.13	95.11	95.11
DenseNet201	94.97	94.98	94.97	94.97
VGG16	95.55	95.56	95.55	95.55
MobileNetV2	96.26	96.27	96.26	96.27
inception_v3i	93.25	93.25	93.25	93.24
The proposed model	98.48	98.56	98.48	98.48

**Table 10:** Comparing previous studies with the proposed model using COVID-19-Symptoms-2.

Papers	The Best Models	Performance
[23]	GRU	A = 98.65, R = 98.6, P = 99.2, and F1 = 99.2
[25]	KNN	A = 97.97, R = 97.97, P = 97.97, and F1 = 97.97
The proposed model	Stacking SVM	A = 99.30, R = 99.30, P = 99.30, and F1 = 99.31

**Table 11:** The proposed model is compared to recent studies using COVID-19-chest-X-ray-2.

Papers	Models	Image Classes	Performance
[28]	ResNet50	Normal COVID-19 pneumonia	A = 98 and R = 96.46
[29]	COVID-Net	Normal COVID-19 pneumonia	A = 92.4
[30]	Concat_CNN	Normal COVID-19 pneumonia	A = 96.31, P = 95.8, and R = 92.99
[32]	Concatenated CNN model	Normal COVID-19 pneumonia	A = 98.02, F1 = 98.24, P = 97.04, R = 98.49
[34]	SqueezNet	Normal COVID-19 pneumonia	A = 95, P = 94.66, R = 94.66, and F1 = 94.66
[3]	XGBoost	Normal COVID-19 pneumonia	A = 97.87, P = 97.87, and R = 98.93
The proposed model	Stacking ensemble DL	Normal COVID-19 pneumonia	A = 99.62, P = 99.66, R = 99.62, and F1 = 99.62
The proposed model	Stacking ensemble DL	Normal COVID-19 pneumonia	A = 98.48, P = 98.56, R = 98.48, and F1 = 98.48

## Data Availability

All datasets used to support the findings of this study are available from the direct link in the dataset citations.

## Abbreviations

The following abbreviations are used in this manuscript:

Artificial intelligence	AI
Machine learning	ML
Deep learning	DL
Convolutional neural network	CNN
Gated recurrent unit	GRU
Long short-term memory	LSTM
Recurrent neural network	RNN
Support vector machine	SVM
k-nearest neighbors	KNN
Random forest	RF
Multilayer perceptron	MLP
XGBoost	XGB
Naive Bayes	NB
Decision tree	DT
LSTM-Layer1	One-layer LSTM
LSTM-Layer2	Two-layer LSTM
GRU-Layer1	One-layer GRU
GRU-Layer2	Two-layer GRU
RNN-Layer1	One-layer RNN
RNN-Layer2	Two-layer RNN
MLP-Layer1	One hidden layer
MLP-Layer2	Two hidden layers
Visual geometry group	VGG
Deep residual networks	ResNet
Accuracy	A
Recall	R
Precision	P
F1-score	F1
